# Optimization and Evaluation of Ventilation Mode in Marine Data Center Based on AHP-Entropy Weight

**DOI:** 10.3390/e21080796

**Published:** 2019-08-15

**Authors:** Guozeng Feng, Shuya Lei, Yuejiao Guo, Bo Meng, Qingfeng Jiang

**Affiliations:** School of Energy and Power, Jiangsu University of Science and Technology, Zhenjiang 212003, China

**Keywords:** ventilation mode, AHP-entropy weight, CFD, marine data center

## Abstract

The ventilation mode affects the cooling efficiency of the air conditioners significantly in marine data centers. Three different ventilation modes, namely, underfloor ventilation, overhead ventilation, side ventilation, are numerically investigated for a typical marine data center. Four independent parameters, including temperature, velocity, air age, and uniformity index, are applied to evaluate the performances of the three ventilation modes. Further, the analytic hierarchy process (AHP) entropy weight model is established and further analysis is conducted to find the optimal ventilation mode of the marine data center. The results indicate that the underfloor ventilation mode has the best performance in the airflow patterns and temperature distribution evaluation projects, with the highest scores of 91.84 and 90.60. If low energy consumption is required, it is recommended to select the overhead ventilation mode with a maximum score of 93.50. The current evaluation results agree fairly well with the three dimensional simulation results, which further proves that the AHP entropy weight method is reasonable and has a high adaptability for the evaluation of air conditioning ventilation modes.

## 1. Introduction

In recent decades, numerous data centers have been built throughout the world due to the need for the development of integrated information. It has been reported that power consumption in data centers accounts for approximately 1.3% of the total worldwide electricity consumption in 2010 [1,2]. With the increase of the servers’ heat load, a corresponding higher heat dissipation is required in data centers as the electrical power supplied to the servers is in essence converted to heat [1]. Thus, sufficient cooling has to be provided to ensure the servers’ reliability. Currently, air cooling is still the predominant method for data centers. The indoor air flow distribution has a major impact on the thermal environment in marine data centers and can also greatly affect air cooling energy efficiency [3,4]. Both the recirculation of heated air and a short circuit or a bypass of cold air contribute to insufficient cooling of marine data centers [5,6]. Therefore, the key to guarantee the reliability of equipment operations is that the air flow pattern distributes properly throughout the data centers [7].

Recent studies have proposed some useful strategies to ensure cooling efficiency. The optimization of the indoor ventilation mode is generally considered as an effective solution for airflow management to improve the thermal environment with minimal energy [8,9]. Wibron et al. [10] proposed the aisle containment as a strategy to avoid the mixing of hot and cold air. Alkharabsheh et al. [11] performed a transient analysis on a contained-cold-aisle data center. Yuan et al. [12] studied the effect of the airflow pattern on the cooling efficiency. The results showed that the best temperature distribution was obtained with the air supply angle of 45°. Lu et al. [13] studied the parameters of a rack inlet and exhaust outlet to evaluate air management and cooling performance in a data center. Lu et al. proposed that the fan speed and ventilation rate should be reduced for the sake of humidity control. Cho et al. [3] evaluated a high heat density data center with six types of air distribution systems based on the temperature balance and the flow patterns. The results showed that the overhead distribution showed superior performance in preventing overheating around the server, while the cooling efficiency of the underfloor distribution systems was maximized. Cho et al. [7] proposed an overall index that objectively evaluated the cooling efficiency. They believed that the supply/return air location was an important factor in high-density data centers. Sammakia et.al [14,15] found that some adjustment methods, such as minimizing local hot spots in the data center, affected the air inlet temperatures, which was one of the keys to effective thermal management. These studies took the approach of optimizing the data center layouts and cooling system schemes to achieve energy efficiency and maintain the indoor temperature within the recommended limits [16]. In order to select an optimal air ventilation mode, it is necessary to predict and evaluate the air distribution of the data center during the early design phase.

Although many studies have been devoted to various types of air management, little information is available on a quantitative evaluation of the parameters in the indoor airflow organization. Moreover, most investigations typically utilize a single indicator or qualitative analysis which cannot provide overall assessment results [17]. C.E. Shannon proposed the concept of information entropy in 1948 [18]. The information entropy theory is a comprehensive method based on probability and numerical statistics. On the basis of the information entropy theory, the entropy weight method is proposed to reflect the relative strength of each index in the interaction, and it has been widely applied to multi-factor problems, such as mountain landslide prediction [19], agriculture [19], environment [20], business decision-making [21]. These studies proved that the entropy weight method can evaluate the program scientifically.

The analytic hierarchy process (AHP) is a decision-making tool developed in the 1970s [22]. It integrates multiple independent factors into a composite factor to simplify the complexity of rankings and has been accepted by decision makers [23]. The AHP has also been successfully applied in hospital site selection [24], the evaluation of energy production technologies [25], teaching quality assessment [26], environmental and economic assessment [27].

The entropy weight method could reflect the index’s utility value with a high correlation. The AHP has the ability to handle qualitative indices and build decision making processes systematically [28]. Thus, the merger of the AHP and the entropy weight method could be used in multivariate assessment problems with relative independent indicators. The AHP entropy method has been gradually accepted and recognized [29,30].

This paper aims to establish a comprehensive evaluation model for the ventilation mode based on the AHP entropy weight method. Firstly, three dimensional simulations are performed to investigate the three ventilation modes of the data center. Secondly, the subjective and objective information are fully integrated in the evaluation system. Finally, the optimal mode for each target is chosen according to the total score. As a single scheme may show both advantages and disadvantages, it is impossible to rely solely on subjective judgment or experience to make correct decisions. Furthermore, a wrong decision may eventually lead to equipment failure in actual operation [31]. Therefore, a comprehensive assessment approach, which combines the impact of all indicators, is required to select the most sensible solution. The AHP entropy weight method used in this study provides a theoretical reference for the selection and optimization of air conditioning ventilation modes in the future.

## 2. Geometry and Optimization Approach

**Figure 1:**
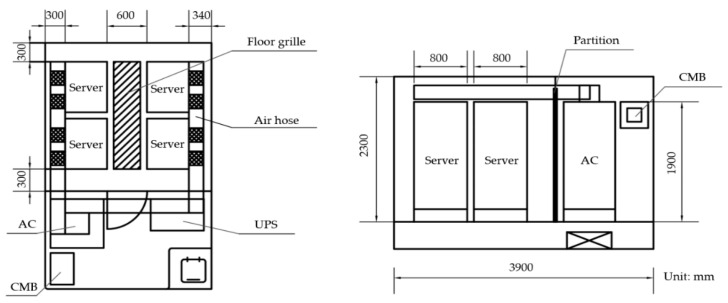
Layout of the data center.

The main computational area of this study is based on drawings provided by AVIC Dingheng Shipbuilding Co., Ltd. (Jiangdu Yanjiang Development Zone, Jiangsu Province, China), which is a marine data center measuring 2.5 m long, 3.9 m wide and 3.6 m high (including sinking floors). Four servers, an air conditioner (AC) with 10 kW power, a UPS power distribution cabinet (UPS), and a comprehensive monitoring box (CMB) were arranged in the domain. Figure 1 shows the layout of the marine data center. To simplify the numerical model and reduce the simulation duration, all devices are treated as cuboids boxes.

**Figure 2:**
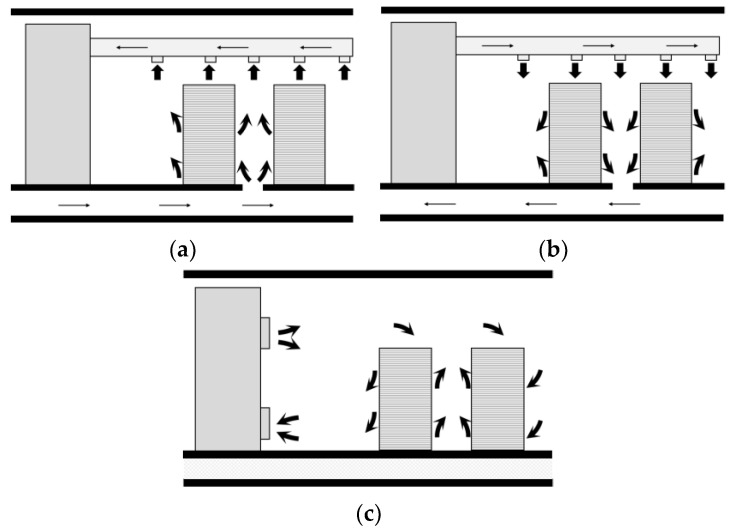
Three air supply modes. (**a**) Case I: underfloor ventilation; (**b**) Case II: overhead ventilation; (**c**) Case III: side ventilation.

The primary goal of the data center air distribution systems is to prevent IT equipment from overheating, thus it is important that the air from the inlet and outlet does not mix and short. The three common ventilation modes mentioned in Rasmussen’s study [32] are shown in Figure 2. In the underfloor ventilation mode, the cold air is blown from the grille on the ground, as shown in Figure 2a. The underfloor ventilation system beneath the raised floor provides conditioned air through diffusers. This ventilation cooling system typically creates vertical temperature stratification, which has an impact on energy, indoor air quality and thermal comfort [3]. As shown in Figure 2b, in the overhead ventilation mode, the cold air is supplied by an air supply duct installed at the top and the heated air is discharged by the floor grille after exchanging heat with the equipment. Figure 2c presents the side ventilation mode. The diffuser and exhaust grille are both mounted on the side wall. The air is sent out from the upper side and discharged on the lower side in this ventilation mode.

## 3. Simulation Method

The computational fluid dynamics (CFD) model of this marine data center was generated in Airpak 3.0.16 (ANSYS, Pittsburgh, PA, USA), which is widely used in analyzing indoor environments with HVAC systems [33,34]. It has been assumed that both the heat dissipation rate and the convective heat transfer coefficients are set as constants during the simulation [35].

The cold air supply in the data center involves the interaction of high quality flow rates with complex thermal fluids [14]. The governing equations of fluid flow describing the property fields can be written as follows:

Continuity:

(1) $$\frac{\partial \rho }{\partial t}+\vec{\nabla }\cdot \left(\rho \vec{u}\right)=0$$

For the three-dimensional steady state flow in this study, the equation can be simplified to:

(2) $$\frac{\partial u}{\partial x}+\frac{\partial v}{\partial y}+\frac{\partial w}{\partial z}=0$$

Conservation of Momentum:

(3) $$\rho \frac{\partial \vec{u}}{\partial t}+\rho \vec{u}\cdot \vec{\nabla }\vec{u}=-\vec{\nabla }P+\vec{\nabla }\tau +\left(\rho -\rho_{\infty }\right)g$$

Similarly, the equation can be simplified to:

(4) $$\rho \left(\frac{\partial u}{\partial x}u+\frac{\partial u}{\partial y}v+\frac{\partial u}{\partial z}w\right)=\left(\frac{\partial \sigma_{x}}{\partial x}+\frac{\partial \tau_{xy}}{\partial y}+\frac{\partial \tau_{xz}}{\partial z}\right)+f_{x}$$

(5) $$\rho \left(\frac{\partial v}{\partial x}u+\frac{\partial v}{\partial y}v+\frac{\partial v}{\partial z}w\right)=\left(\frac{\partial \sigma_{y}}{\partial y}+\frac{\partial \tau_{yx}}{\partial x}+\frac{\partial \tau_{yz}}{\partial z}\right)+f_{y}$$

(6) $$\rho \left(\frac{\partial w}{\partial x}u+\frac{\partial w}{\partial y}v+\frac{\partial w}{\partial z}w\right)=\left(\frac{\partial \sigma_{z}}{\partial z}+\frac{\partial \tau_{zy}}{\partial y}+\frac{\partial \tau_{zx}}{\partial x}\right)+f_{z}$$

where,

(7) $$\sigma_{i}=-p+2\mu \frac{\partial u_{i}}{\partial x_{i}}$$

(8) $$\tau_{ij}=\mu \left(\frac{\partial u_{i}}{\partial x_{j}}+\frac{\partial u_{j}}{\partial x_{i}}\right)$$

Conservation of Energy:

(9) $$\rho \frac{\partial e}{\partial t}+\rho u\cdot \vec{\nabla }e=\nabla \cdot \left(\lambda \nabla T\right)-P\left(\nabla \cdot u\right)$$

(10) $$\rho \frac{De}{DT}=-div\left(\rho \vec{u}\right)+\left[\begin{array}{c} \frac{\partial \left(u\tau_{xx}\right)}{\partial x}+\frac{\partial \left(u\tau_{zy}\right)}{\partial y}+\frac{\partial \left(u\tau_{xz}\right)}{\partial z}\\ +\frac{\partial \left(v\tau_{xx}\right)}{\partial x}+\frac{\partial \left(v\tau_{zy}\right)}{\partial y}+\frac{\partial \left(v\tau_{xz}\right)}{\partial z}\\ +\frac{\partial \left(w\tau_{xx}\right)}{\partial x}+\frac{\partial \left(w\tau_{zy}\right)}{\partial y}+\frac{\partial \left(w\tau_{xz}\right)}{\partial z} \end{array}\right]+div\left(kgradT\right)+S_{E}$$

where, *div* is mathematical operator. *e* is specific internal energy, Q/M. *g* is gravitational acceleration, *M/t^2^*. grad is a mathematical operator. *i*, *j* are symbols that denote any of the space coordinate subscripts. *k* is thermal conductivity, *Q/tLT*. P is system pressure, *F/M^2^. S_E_* is the source term. *u* is velocity in *x* direction, *M/t. v* velocity in *y* direction, *M/t. w* velocity in *z* direction, *M/t. x* is a space coordinate system in *x* direction, *L*. *y* is a space coordinate system in *y* direction, *L*. *z* is a space coordinate system in *z* direction, *L*.

**Figure 3:**
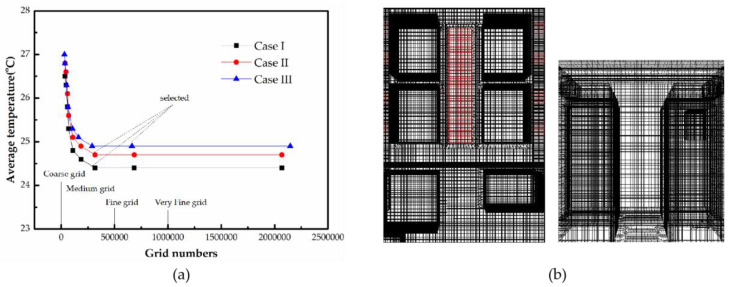
Calculation domain analyses. (**a**) grid independence test results; (**b**) calculation domain.

The realizable *k-ε* turbulence model was applied in this study to account for the turbulence. The grid independence analysis was conducted, and eight different grid numbers were applied for each model [36]. The average temperature of the data center was monitored for the grid independence test, as shown in Figure 3a. Figure 3b depicts the grid of the calculation area. It was verified that the optimum mesh for the investigated three models were 317,452, 315,263 and 289,891.

Due to the low air supply speed of the data center air conditioner, the pressure-velocity coupling solver, the SIMPLE algorithm and the second order upwind discrete method were adopted. The convergence criteria for this study is specified as three orders of a magnitude drop in the mass and momentum conservation equations, and five orders in the energy conservation equation [37,38].

The internal wall was set as a partition with a given convective heat transfer coefficient of 2.5 W/(m^2^°C). The servers and other equipment are regarded as blocks with heat sources. The boundary conditions of the three cases are summarized in Table 1. The designed working condition was at the room temperature of 24 °C, air flow velocity of 2 m/s, air age of 15 s. It has been assumed that the ventilated condition is not satisfactory when the temperature deviation exceeds 0.8 °C, the air flow velocity deviation exceeds 1 m/s, or the air age deviation exceeds 3 s.

## 4. Ventilation Index Scoring System

### 4.1. Numerical Analysis of Ventilation Modes

**Figure 4:**
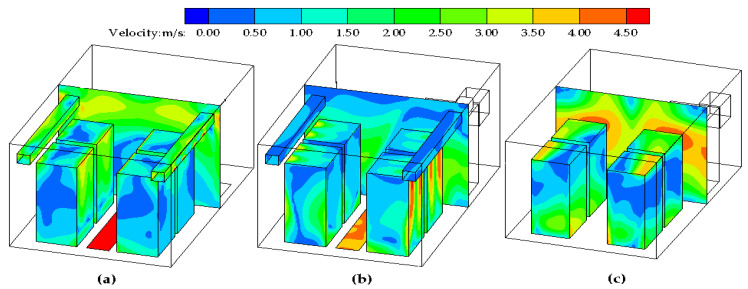
The velocity contours near the server surfaces of the three ventilation modes. (**a**) Case I; (**b**) Case II; (**c**) Case III.

Figure 4 shows the velocity distributions near the surfaces of the servers in the data center. It was found that the airflow velocities near the air inlets and outlets were larger than in the other areas under the three ventilation modes. Figure 4c illustrates that the air in the upper part of the server flows fast, indicating that the convective heat transfer intensity is greater in case III ventilation mode. However, there is almost no air flow between the servers. The hot server surface is not in contact with the cold air. Therefore, it is not conducive to eliminate the heat dissipation in the marine data center. However, in the other two ventilation modes, as shown in Figure 4a,b, the air velocities are comparatively large near the surfaces of the servers.

**Figure 5:**
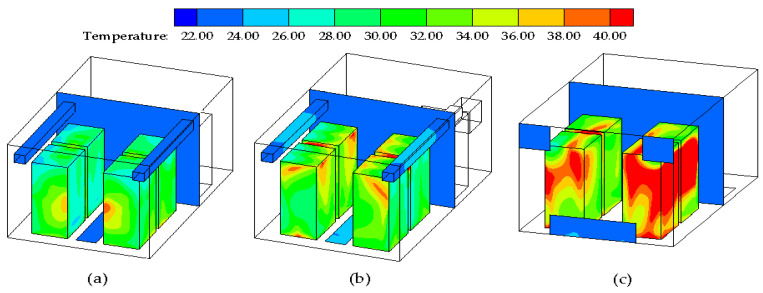
Temperature distributions of three air supply modes. (**a**) Case I; (**b**) Case II; (**c**) Case III.

Figure 5 shows the temperature distributions under three ventilation modes. As shown in Figure 5a, a good temperature distribution is shown in case I as the local hotspot area of the data center is the smallest. Moreover, the room temperature is the lowest and closest to the design temperature of 24 °C. As shown in Figure 5b, the supply air in case II does not effectively bring away the heat generated at the lower section of the server compared with case I. Figure 5c shows that the large areas of high temperatures are distributed near the servers surfaces, which means that the cold air is unable to dissipate heat in this ventilation mode. This may be due to insufficient airflow cycling and inadequate convective heat exchange with the equipment. It should be noted that the contours plotted based on the results of the numerical simulations analyze the temperature field from an intuitive and qualitative perspective. Therefore, quantitative analysis of the temperature distribution is necessary.

**Figure 6:**
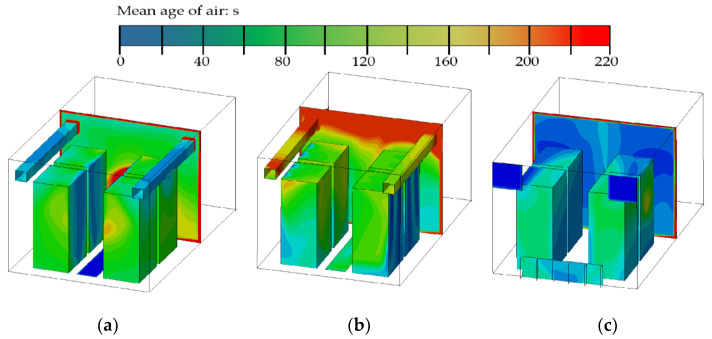
The air age distributions of the three air supply modes. (**a**) Case I; (**b**) Case II; (**c**) Case III.

Figure 6 shows the air age distributions under the three ventilation modes. The air age, i.e., the age of the air mass point refers to the time in which the air stays in the room. It reflects the freshness of the indoor air which can comprehensively measure the ventilation effect of the room. It is an important indicator for evaluating indoor air quality [39]. The average air ages of the central section under the three ventilation modes are 111 s, 142 s, and 86.9 s, respectively. Figure 6c indicates the overall air age is too small in case III. Although the side ventilation mode can shorten the contact time between the cold airflow and the servers, it reduces energy utilization. However, it is difficult to judge the pros and cons of case I and case II just based on the contours of Figure 6a,b. Therefore, it is necessary to use the heat removal efficiency as an additional evaluation index for further comparison. The calculation results are shown in Table 2.

**Figure 7:**
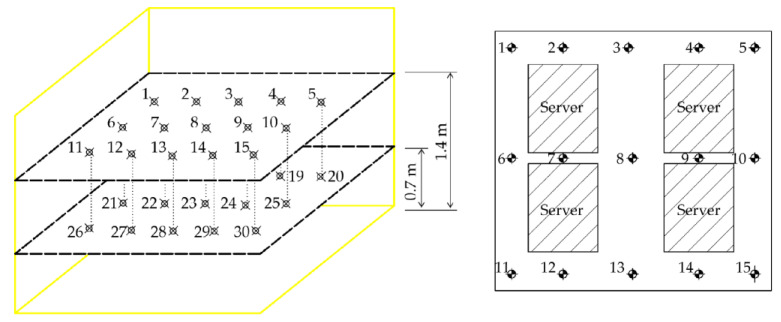
Monitoring point layout.

In order to further analyze the flow field of the data center quantitatively, thirty measure points were fixed to monitor the value of the velocities and temperatures based on the above simulation results [40]. As shown in Figure 7, the points 1~15 are at the horizontal plane of *z* = 0.7 m, and the points 16~30 are at the horizontal plane of *z* = 1.4 m.

**Figure 8:**
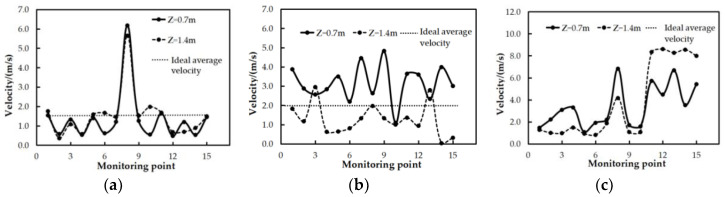
The velocity distribution of monitoring points. (**a**) Case I; (**b**) Case II; (**c**) Case III.

**Figure 9:**
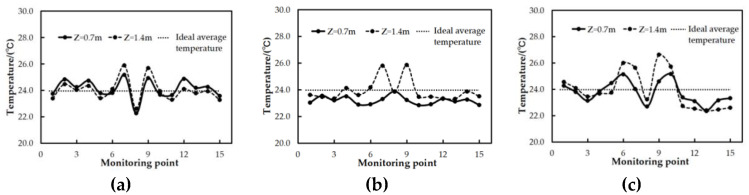
The temperature distribution of monitoring points. (**a**) Case I; (**b**) Case II; (**c**) Case III.

From the calculation results of the uniformity, the local high temperature region of the marine data center can be accurately found, as shown in Figure 8 and Figure 9. Figure 8a shows that a fair velocity distribution of air is observed in most parts of the marine data center under case I, except that the air velocity is higher near the air outlet. Figure 8b,c indicate that the velocity distributions are discrete in the latter two modes of the air supply, especially in case III. It was found in Figure 9 that temperature uniformity of the thirty monitoring points was evenly distributed within ideal operating conditions under case I and case II. From Figure 9, it can be seen that the temperature fluctuations in case I are the smallest while in case III, they are the largest.

### 4.2. Ventilation Index Scoring Method

The percentage *P_i_*of the sample whose characteristic parameters exceed the design requirements can be calculated as follows:

(11) $$P_{i}=\frac{N_{C}}{N}$$

where, *N_C_* is the number of samples whose temperature exceeds the design requirements; *N* is the total number of samples.

If *P_i_* ≤ 5%, *S_i_* = 100%; *P_i_* > 5%, the satisfaction *S_i_* is defined by the following equation:

(12) $$S_{i}=100\times \exp\left\{-\left\{0.03353\times {\left[2\times \left(P_{i}-0.05\right)\right]}^{4}+0.2179\times {\left[2\times \left(P_{i}-0.05\right)\right]}^{2}\right\}\right\}$$

where *i* takes 1, 2, 3, which represents the three characteristic parameters of the temperature, airflow velocity and air age, respectively; *S_i_* is the satisfaction degree of factor *i*.

Weltens proposed the concept of the uniformity index based on the statistical deviation definition and CFD prediction in 1993 [41]. The method can quantitatively reflect the flow velocity uniformity on the velocity section, and thus is widely used in the flow field analysis of liquids and gases. The uniformity index is calculated using the equation below:

(13) $$S_{\gamma }=100\times \left(1-\frac{1}{2n}\overset{n}{\underset{j=1}{\sum }}\frac{\sqrt{{\left(v_{j}-\bar{v}\right)}^{2}}}{\bar{v}}\right)$$

where, *S_Υ_* is the index score of uniformity; *ν_j_* is the velocity value of the *j_th_* collection point; $\bar{v}$ is the average velocity; *n* is the number of sampling points, *n* = 1, 2, …30.

The heat removal efficiency score (HRE) is described as follows:

(14) $$y=100\times \left[1-e^{-2\left(x-0.2\right)}\right]$$

where *y* represents the score of the heat removal efficiency; *x* represents the heat removal efficiency, which reflects the ability of the ventilation system to eliminate excess heat, and it can be illustrated as below [42]:

(15) $$x=\frac{t_{e}-t_{s}}{t_{p}-t_{s}}$$

where *t_e_* is the return air temperature; *t_s_* is the supply air temperature; *t_p_* is the temperature of the measuring point.

An initial decision table was created by defining the independent factors as a decision attribute set. The temperature satisfaction, velocity satisfaction, air age satisfaction, uniform satisfaction, and heat removal efficiency satisfaction are listed in Table 2. Case I is superior in the velocity and uniformity satisfaction. Case II has the highest score in terms of the temperature satisfaction, and heat removal efficiency. Although the air age satisfaction of case III is the highest, the heat rejection efficiency satisfaction is the lowest. It can be seen from the calculation results that the three ventilation modes have their own advantages and disadvantages. Therefore, the optimal ventilation mode cannot be determined just based on the satisfaction of individual indicators.

## 5. Evaluation for Three Ventilation Modes

### 5.1. Build the AHP-Entropy Weight Model

**Figure 10:**
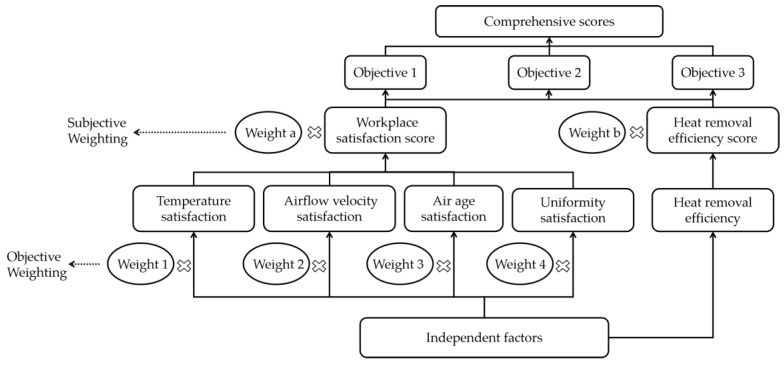
The evaluation process of the analytic hierarchy process (AHP)-entropy weight method.

In this study, the entropy weight method was combined with the AHP concept to evaluate the air distributions in the marine data center. The temperature satisfaction, airflow velocity satisfaction, air age satisfaction, and uniformity satisfaction were all integrated into the work area satisfaction. Moreover, the work area satisfaction and heat recovery efficiency satisfaction were further combined into one final decisive evaluation index, from which the optimal solution can be obtained directly. Figure 10 depicts the evaluation process of the AHP entropy weight method.

### 5.2. Define Subjective Weight

Due to the different focus of the design goals, the weight requirements for the factors are also different. This method of assigning weights based on the design goals is also called subjective weighting, which is usually defined by design experience. The subjective weight allocation scheme for the workplace satisfaction score and the heat efficiency score are listed in Table 3.

### 5.3. Define Objective Weight

As independent factors are not directly related to the design goals, it is necessary to calculate the weights of the independent factors based on the information entropy. The weights of the four independent factors are calculated according to the following steps:

Formally, a matrix *M_m×n_* = (*X*1, *X*2*,*… *X_k_*) is set as an information system, where *X_i_* = (*x*1, *x*2, *x*3*,*… *x_n_*) [18]. *X_i_* is standardized as:

(16) $$Y_{ij}=\frac{x_{ij}-min\left(x_{i}\right)}{max\left(x_{i}\right)-min\left(x_{i}\right)}$$

(17) $$I_{ij}=\frac{Y_{ij}}{\sum_{j=1}^{n}Y_{ij}}$$

where *I_ij_* is the probability value of *x_ij_*, indicating the contribution degree of the *j*th factor under the *i*th case.

The information entropy of each factor (*E_j_*) is calculated by the following equation:

(18) $$E_{j}=-ln{\left(n\right)}^{-1}\overset{n}{\underset{i=1}{\sum }}I_{ij}lnI_{ij}$$

where if $I_{ij}=0$, $\underset{p_{ij}\to 0}{\lim}I_{ij}lnI_{ij}=0$.

In decision-making systems, the weight of the conditional attribute *W_i_* can be calculated as:

(19) $$W_{i}=\frac{1-E_{i}}{k-\sum E_{i}}$$

According to the previous calculation results, the scoring matrices *X_ij_*, probability value *I_ij_*, information entropy *E_ij_*, and entropy weights *W_i_* of the four indicators are as follows.

(20) $$X_{ij}=\left[\begin{array}{c} \begin{array}{ccc} 99.73 & 98.14 & 99.42\\ 72.30 & 89.19 & 20.85\\ 92.37 & 89.53 & 93.46 \end{array}\\ \begin{array}{ccc} 75.31 & 75.00 & 66.86 \end{array} \end{array}\right],\;I_{ij}=\left[\begin{array}{cc} \begin{array}{cc} 0.52 & 0.55\\ 0.48 & 0.45\\ 0 & 0 \end{array} & \begin{array}{cc} 0.42 & 0.51\\ 0 & 0.49\\ 0.58 & 0 \end{array} \end{array}\right],$$

(21) $$E_{i}=\left[\begin{array}{cc} \begin{array}{cc} 0.630 & 0.627 \end{array} & \begin{array}{cc} 0.619 & 0.631 \end{array} \end{array}\right],\;W_{i}=\left[\begin{array}{cc} \begin{array}{cc} 0.248 & 0.250 \end{array} & \begin{array}{cc} 0.255 & 0.247 \end{array} \end{array}\right]$$

Generally speaking, the smaller the information entropy is, the greater the fluctuation of the index value is, and the more information is provided, and the larger the weight is.

The work area satisfaction scores (WS) were calculated as follows:

$$WS_{j}=W_{i}X_{ij}=\left[\begin{array}{ccc} 89.86 & 88.61 & 84.44 \end{array}\right]$$

The four predictors were compared with each other to determine their relative importance. The matrix of the weights *W_i_* presents that air age satisfaction is a crucial predictor for the workplace satisfaction score with the largest weight of 0.255. The score of the work area satisfaction are 89.89, 88.65, 88.4 respectively. It shows that the work satisfaction of case I is the highest. Similarly, the final score of the comprehensive evaluation index can be obtained.

### 5.4. Evaluation Results

**Figure 11:**
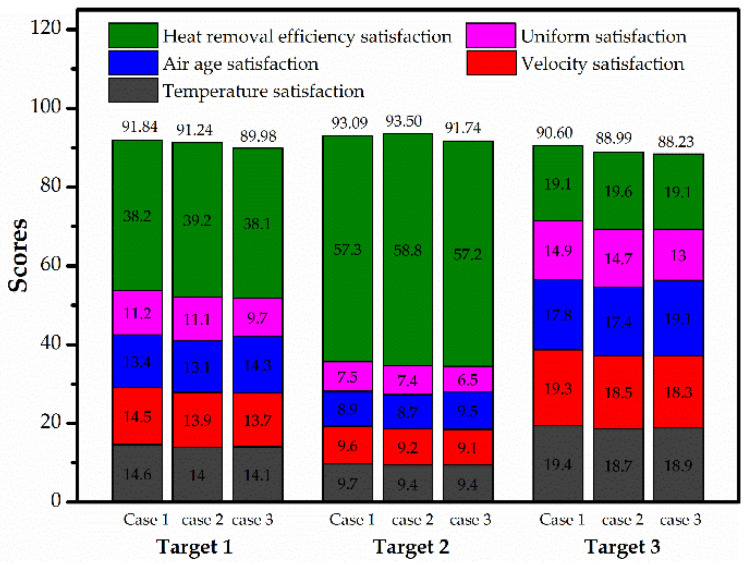
The evaluation results based on the AHP entropy weight method.

The priorities and alternatives of air conditioning schemes can be determined according to the evaluation results to allocate resources and requirement. Figure 11 shows the evaluation results based on the AHP entropy weight method under the three different design targets. It is presented that case I has the best performance with the highest scores in both Schemes 1 and 2 (91.84 and 90.60), and case II ranks the second. If low energy consumption is required, it is recommended to select case II with a maximum score of 93.50, followed by case I. Case III scores the lowest under the three design goals. The model can fully integrate the subjective and objective information in the process of evaluation. The evaluation results agree fairly well with the simulation results.

## 6. Conclusions

In marine data centers with high-density heat sources, local hotspots and large energy costs are caused by uneven air distributions and poor air management. To investigate the effects of the supply and return air location on the cooling efficiency, three types of ventilation modes were established and numerically simulated. The AHP entropy weight evaluation system was proposed to assign weights for the important factors such as the temperature, velocity, air age, and uniformity satisfaction. The optimal ventilation mode was selected by the final evaluation scores. The main conclusions can be drawn:

(1) If a more uniform airflow and higher indoor air quality are required, it is better to choose the underfloor ventilation mode, which has the highest overall score of 91.84 and 90.60 respectively.
(2) When lower energy consumption is required, the overhead ventilation mode should be selected with a maximum score of 93.50. This ventilation mode shows superior performance in both the temperature and uniformity satisfaction.
(3) The AHP entropy weight method is successfully applied to the optimization of the air conditioning ventilation mode in the marine data center. The evaluation model presented in this study may contribute to the practical application and selection of other air conditioning systems.

## Figures and Tables

**Table 1:** Summary of the boundary conditions used in computational fluid dynamics (CFD simulations).

Boundaries		Parameters		
		Case I	Case II	Case III
Openings	Inlets	*V_z_* = 2 m/s	*V_z_* = −2 m/s	*V_x_* = 2 m/s
		*T_in_* = 22 ℃, *Q*_in_ = 3800 m^3^/h,		
	Outlet	*T_out_* = 26 ℃		
Walls	Internal wall	*H* = 2.5 W/(m^2^℃)		
	Floor and ceiling	Adiabatic walls		
Heat sources	Server	3000 W		
	AC	10 kW		
	UPS	500 W		
	CMB	300 W		

**Table 2:** The index satisfaction of the three air supply modes.

Air Supply Mode	T-S ^1^	V-S ^2^	A-S ^3^	U-S ^4^	HES ^5^
Case I	97.96	93.66	92.37	75.31	95.58
Case II	97.29	92.53	89.53	75.00	98.02
Case III	89.99	87.11	93.46	66.86	95.26

^1^ Temperature satisfaction; ^2^ Velocity satisfaction; ^3^ Air age satisfaction; ^4^ Uniform satisfaction; ^5^ Heat removal efficiency satisfaction.

**Table 3:** The independent factor subjective weight allocation scheme.

Design Scheme		Work Area Satisfaction Score	Heat Efficiency Score
Scheme 1	Meet work area requirements	0.6	0.4
Scheme 2	Save air conditioning energy	0.4	0.6
Scheme 3	Keep the air clean	0.8	0.2

## Nomenclatures

*div*	mathematical operator	*t*	Time (s)
*e*	specific internal energy (J)	*t_s_*	Supply air temperature (℃)
*F*	External bulk force (N)	*t_e_*	Return air temperature (℃)
*g*	Gravitational force (m^2^/s)	*t_p_*	Temperature of the measuring point
*h*	Convective heat transfer coefficient of internal partition (W/(m^2^℃))	*T_in_*	Air inlet temperature (℃)
*I_ij_*	Probability value of *x_ij_,* indicating the contribution degree of the *j*th factor under the *i*th case.	*T_out_*	Air outlet temperature (℃)
*J_j_*	Diffusion flux of component *j* (kg/(m^2^s))	*u*	velocity in *x* direction (m/s)
*k*	thermal conductivity	*v*	velocity in *y* direction (m/s)
*N*	Total number of samples	*V_x_*	Velocity in the x direction (m/s)
*N_C_*	Number of samples whose temperature exceeds the design requirements	*V_z_*	Velocity in the z direction (m/s)
*n*	Number of sampling points	*ν_j_*	Velocity value of the *j* th collection point (m/s)
*P*	Pressure (Pa)	$\;\bar{v}$	Average velocity (m/s)
*P_i_*	Percentage of the sample whose characteristic parameter exceeds the design requirements (%)	*W_i_*	Weight of the conditional attribute
*Q_in_*	Air flow rate in air inlet (m^3^/h)	*w*	velocity in *z* direction (m/s)
*S_i_*	Satisfaction degree of factor *i*	*x*	space coordinate system in *x* direction
*S_Υ_*	Index score of uniformity	*y*	space coordinate system in *y* direction
*S_E_*	source term	*z*	space coordinate system in *z* direction
**Greek Symbols**			
*ρ*	Density (kg/m^3^)	*τ*	Shear stress (N)
▽	mathematical operator	*μ*	dynamic viscosity
*σ*	stress tensor	*λ*	thermal conductivity
